# Successful Treatment of Tourette Syndrome With a Combination of Guanfacine and Aripiprazole: A Case Series

**DOI:** 10.7759/cureus.39573

**Published:** 2023-05-27

**Authors:** Tikku George, Mallory Emig, Pratap Chand, Heer Hendry

**Affiliations:** 1 Neurology, Saint Louis University School of Medicine, Saint Louis, USA

**Keywords:** aripiprazole, guanfacine, vocal tic, motor tic, tourette's syndrome

## Abstract

Tourette syndrome (TS) is a disorder of the nervous system that causes motor and vocal tics. Tics occur as sudden onset, rapid stereotyped purposeless movements or sounds. Combination therapies can be utilized for adequate control of motor and vocal tics. Patients diagnosed with TS and treated with aripiprazole and guanfacine from 2011-2022 at Saint Louis University Hospital were retrospectively surveyed. Three patients with TS treated with aripiprazole and guanfacine experienced significant improvement or complete resolution of their motor and vocal tics. In our cohort of three patients, the combination of guanfacine and aripiprazole significantly improved or resolved motor and vocal tics that were previously poorly controlled on other traditional medications.

## Introduction

Tourette syndrome (TS) is a childhood-onset neuropsychiatric disorder involving multiple motor and vocal tics [1]. Tics can occur as sudden onset, rapid stereotyped purposeless movements, or sounds and are often accompanied by psychiatric co-morbidities including obsessive-compulsive disorder (OCD), attention deficit hyperactivity disorder (ADHD), depression, and anxiety [2]. The co-existence of these co-morbid conditions can further complicate the attempts to find suitable treatment options. Despite the treatment options available for tic disorders, many patients’ tics remain poorly controlled by traditional therapies. Combination therapies are often utilized for the control of motor tics.

The overall prevalence of TS is estimated to be around 1% of the population [3]. The prevalence of tic disorders is more difficult to estimate, but some estimates indicate up to one in five children experience some form of transient tic during their childhood [4]. Even though these disorders represent a small percent of the adult population, they cause significant psychosocial distress and can lead to significant mental and physical distress in this patient population. Tics specifically present with sudden onset, intermittent movements, or phonic activity which range in severity and may lead to significant impairment of a patient’s daily functioning and quality of life.

Although there is no cure for TS, treatment is multimodal with pharmacotherapy, behavioral therapies, and psychoeducation. Risperidone, clonidine, and aripiprazole are the most commonly prescribed medications [3]. However, they are not known to cause complete resolution of symptoms. Risperidone, in one clinical trial, showed a 32% reduction in tic severity compared to placebo [5]. Even though a combination of therapies is often used to control tic disorders including TS, the specific combination of guanfacine and aripiprazole has not been systematically studied. We describe the clinical response to guanfacine and aripiprazole in three patients with TS.

## Case presentation

Case 1

A 56-year-old male with a history of TS from childhood presented to our clinic. He had been trialed on multiple medicines in the past including pimozide and haloperidol. Upon presentation, he was on treatment with clonidine 0.1 milligrams (mg) per day, which had provided no improvement in his tics. In the two years prior to presentation, the patient stated that the tics were becoming more frequent. Jerking of his shoulders resulted in rib fractures which had since healed. Co-morbid conditions included bulimia and ADHD which were treated with dextroamphetamine-amphetamine and bupropion. 

Examination revealed an anxious affect and diaphoresis on the forehead. No vocal tics were noted. He had near-continuous motor tics consisting of shrugging of the shoulders and jerks affecting the trunk and abdomen. No other neurologic deficits were noted. He underwent a magnetic resonance imaging (MRI) scan of the brain two years prior which was unremarkable. 

The patient was started on aripiprazole 5 mg at bedtime and guanfacine 1 mg at bedtime. The clonidine was stopped. He was referred to behavioral therapy for habit reversal techniques for tic disorders. At the three-month follow-up, the patient stated that both his vocal and motor tics had resolved completely. No tics were noted during the examination and he denied adverse effects from the medications. He was followed up at six-month intervals for three years via telemedicine and his tics remained resolved. He continued to deny medication adverse effects and was content with the results the medications had provided.

Case 2

A 19-year-old female patient with a history of depression presented with 14 months of motor and vocal tics. These symptoms worsened after initiation of escitalopram for depression three months prior to presentation. Her motor tics involving her hands, arms, and legs would occur throughout the day. These tics consisted of jerking movements that had led to minor injuries from striking nearby objects. Vocal tics consisted of saying words she did not mean to while speaking as well as echolalia. She also had vocalizations of grunting or screaming but denied coprolalia or copropraxia. She endorsed an urge to do these movements with the initial ability to suppress the urge. However, there was then a buildup of tension forcing her to perform the movements. 

She was started on clonazepam 0.5 mg three times daily which did not improve her tics, so it was stopped. Her primary care physician started her on carbidopa/levodopa 25/100 mg 0.5 tablets three times per day and melatonin without any benefit. On initial examination, she displayed multiple, diffuse motor tics and dystonic movement of the right third finger. She was started on risperidone 0.5 mg twice a day which improved her tics by about 50% according to the patient. 

Subsequent examination in the outpatient neurology clinic demonstrated multiple involuntary movements and vocal sounds including clicking her tongue, grunting, and motor tics of hands with quick flexion of the right index finger. She would often hit her shoulder with her hands. 

She was diagnosed with TS with vocal and motor tics with motor tics of sufficient amplitude to produce injury. The diagnosis of TS at the age of 19 was uncommon and the differential of pediatric acute-onset neuropsychiatric syndrome was also considered. The patient denied psychosocial stressors and illicit substance use. Blood chemistries including serum ceruloplasmin were normal. The Streptococcus swab was negative and antistreptolysin (ASO) titer to screen for group A streptococcus exposure was within normal limits. She was given a course of azithromycin which did not improve her tics. MRI of the brain was normal. 

Risperidone 1 mg twice daily was discontinued and replaced with aripiprazole 5 mg at bedtime. Guanfacine 1 mg at bedtime was also started. At her three-month follow-up, the patient stated that with the combination of guanfacine and aripiprazole, the tics had improved by 80%. Her vocal tics of screaming, shouting, and echolalia had completely resolved. She was getting motor tics a few times a week. She denied the adverse effects of the medications. She had started exercising and was doing better in school.

Case 3

A 27-year-old male with a history of vocal and motor tics since the age of nine presented to the clinic. The vocal tics consisted of squeaking noises and the motor tics involved his head, neck, hands, and feet. He was diagnosed at around age 10 with TS. He also had co-morbid OCD, ADHD, anxiety, and depression. He had taken multiple medications over the years without full relief from the tics. This prevented him from both finishing an advanced degree in his field and being able to work. He had presented to us after his tics worsened in severity over the prior four years. Specifically, the dystonic tics affecting the right lateral flexion of the neck and right shoulder had caused cervical disc prolapses and bruising of the shoulder respectively. Hence, his diagnosis was modified to malignant TS.

In the past, he was treated with aripiprazole, but high doses of up to 10 mg per day caused severe akathisia that was partially improved with mirtazapine. He briefly trialed ziprasidone but this also caused akathisia so it was stopped. He was started on a combination of guanfacine 1 mg at bedtime and aripiprazole 2.5 mg daily. The aripiprazole dosage caused some akathisia so it was initially reduced to 1/3rd of a 5 mg tablet and this significantly improved the cervical dystonic tic. He had slight akathisia in the form of restlessness in his feet, but he reported it was manageable and denied other adverse effects. He was able to later increase it to 5 mg daily with continued control of his cervical dystonic tic. 

## Discussion

Two of the three patients experienced the onset of their symptoms in childhood, which is a more classical picture of TS. Both had persistent vocal and motor tics into adulthood. The other patient experienced the onset of symptoms at age 17, which is unusual for the development of TS. All three patients had various co-morbid psychiatric diagnoses that were additional sources of distress. Despite the differences in symptom onset and the unique psychiatric profiles of the patients, all three patients had a successful response to low doses of guanfacine and aripiprazole with one patient experiencing complete resolution of his tics.

Though the exact mechanisms for tic disorders have yet to be determined, alterations in dopaminergic neurotransmission within cortico-striatal-thalamo-cortical circuitries are thought to play a pivotal role in the pathophysiology of TS [6]. Specifically, it has been suggested that tic expression might be linked to a hyperdopaminergic state due to increased receptor sensitivity, tonic phasic dysfunction, and both pre- and post-synaptic dysfunction [7]. Antidopaminergic medications are still considered the most effective pharmacotherapy for tics [8-9]. However, these pharmacotherapeutic agents have different degrees of dose-dependent adverse effects that often limit their use. In children with TS and tic-related OCD, there is evidence to support the use of antidopaminergic augmentation from a case series of children treated with aripiprazole or risperidone [10].

The exact mechanism of action of aripiprazole is still being uncovered, but it has unique properties among the atypical antipsychotics regarding its “adaptive” pharmacologic profile and it can potentially act as a full antagonist, a moderate antagonist or a partial agonist at dopamine D2, D3, D4 receptors as well as 5-HT1A and 5-HT2C serotonergic receptors [6, 11]. Aripiprazole is considered a third-generation antidopaminergic medication. The effectiveness of aripiprazole in reducing tics has been estimated to be at least comparable to other dopamine-modulating agents, such as haloperidol and risperidone [12-13]. The results of a prospective uncontrolled open-label study in adults with TS showed that aripiprazole can significantly improve co-morbid conditions, especially OCD, in addition to tics [14]. The adverse effect profile of aripiprazole is superior to the other antidopaminergic agents used for the treatment of TS [15-16]. Aripiprazole causes less sedation and weight gain; it also has the unique property of potentially reducing serum prolactin concentration [17-18]. With this favorable efficacy-to-tolerability ratio, aripiprazole is at present the most commonly used antidopaminergic medication for the treatment of both tics and co-morbid behavioral symptoms in patients with TS and has superseded risperidone as the first choice anti-tic agent [19-20].

The autonomic nervous system is implicated in the pathophysiology of TS as the tic activity is linked to a patient's psychological and emotional state [17]. Sympatholytic drugs such as clonidine and guanfacine are often used as the first-line medication to treat patients with TS [17]. Both clonidine and guanfacine bind to alpha-2 adrenergic receptors at the pre-synaptic level, resulting in the decreased release of noradrenaline. Alpha-2 agonists have long been used for the treatment of both tics and behavioral symptoms, ranging from attention deficit and hyperactivity disorder to irritability.

Guanfacine is most frequently used to treat ADHD, but it can also be used for tics and TS. It acts as a selective norepinephrine (NE) α2A-adrenoceptor agonist (α2A-AR) and the main area of the brain where this action appears most relevant is the prefrontal cortex (PFC) [17]. The PFC is responsible for higher-order cognitive and executive functions. Guanfacine has been shown to act within the PFC dendrites to inhibit cAMP-PKA-K+ channel signaling, thereby strengthening network connectivity and improving cognitive functions. Evidence of additional α2A-AR actions of guanfacine like weakening plasticity in the amygdala, reducing NE release, and deactivation of microglia with subsequent reduction in inflammation provide additional benefits for stress-related disorders [17].

In our patients, we hypothesize that the combination of guanfacine acting through reduced NE activity and PFC and aripiprazole acting through decreased baseline D2 stimulation and partial receptor agonism at the D2, D3, and D4 dopaminergic receptors acts on two different pathophysiologic targets of TS. The favorable efficacy to adverse effects profile of aripiprazole adds to the benefits of therapy.

Larger groups of patients are needed to determine the true efficacy of the combination of guanfacine and aripiprazole, but the successful outcomes of these patients with previously poorly controlled symptoms on other medications show promise for this unique therapy.

## Conclusions

In our cohort of three patients, the combination of guanfacine and aripiprazole significantly improved or resolved motor and vocal tics that were previously poorly controlled on other traditional medications. Clinicians taking care of patients with chronic tic disorders, including Tourette's syndrome, should consider this combination for effective control of motor and vocal tics.
